# Physical health-related quality of life in relation to metabolic health and obesity among men and women in Germany

**DOI:** 10.1186/s12955-017-0688-7

**Published:** 2017-06-10

**Authors:** Julia Truthmann, Gert B. M. Mensink, Anja Bosy-Westphal, Ulfert Hapke, Christa Scheidt-Nave, Anja Schienkiewitz

**Affiliations:** 1 Department of Epidemiology and Health Monitoring, Robert Koch Institute, Berlin, Germany; 2 Institute of Nutritional Medicine, University of Hohenheim, Stuttgart, Germany

**Keywords:** Metabolically healthy obesity, SF-36, Physical component summary, Sex, Socio-economic status, Lifestyle, Comorbidities, Germany, DEGS1

## Abstract

**Background:**

This study examined sex-specific differences in physical health-related quality of life (HRQoL) across subgroups of metabolic health and obesity. We specifically asked whether (1) obesity is related to lower HRQoL independent of metabolic health status and potential confounders, and (2) whether associations are similar in men and women.

**Methods:**

We used cross-sectional data from the German Health Interview and Examination Survey 2008–11. Physical HRQoL was measured using the Short Form-36 version 2 physical component summary (PCS) score. Based on harmonized ATPIII criteria for the definition of the metabolic health and a body mass index ≥ 30 kg/m^2^ to define obesity, individuals were classified as metabolically healthy non-obese (MHNO), metabolically unhealthy non-obese (MUNO), metabolically healthy obese (MHO), and metabolically unhealthy obese (MUO). Sex-specific analyses including multivariable linear regression analyses were based on PCS as the dependent variable, metabolic health and obesity category as the independent variable with three categories and MHNO as the reference, and age, education, lifestyle and comorbidities as confounders.

**Results:**

This study included 6860 participants (3298 men, 3562 women). Compared to MHNO, all other metabolic health and obesity categories had significantly lower PCS in both sexes. As reflected by the beta coefficients [95% confidence interval] from bivariable linear regression models, a significant inverse association with PCS was strongest for MUO (men: −7.0 [−8.2; −5.8]; women: −9.0 [−10.2; −7.9]), intermediate for MUNO (men: −4.2 [−5.3; −3.1]; women: −5.6 [−6.8; −4.4]) and least pronounced for MHO (men: −2.2 [−3.6; −0.8]; women −3.9 [−5.4; −2.5]). Differences in relation to MHNO remained statistically significant for all groups after adjusting for confounders, but decreased in particular for MUNO (men:–1.3 [−2.3; −0.3]; women: −1.5 [−2.7; −0.3].

**Conclusions:**

Obesity was significantly related to lower physical HRQoL, independent of metabolic health status. Potential confounders including age, educational status, health-related behaviors, and comorbidities explained parts of the inverse relationship. Associations were evident in both sexes and consistently more pronounced among women than men.

**Electronic supplementary material:**

The online version of this article (doi:10.1186/s12955-017-0688-7) contains supplementary material, which is available to authorized users.

## Introduction

Physical health-related quality of life (HRQoL) is a clinically relevant predictor of adverse health outcomes e.g. disability [1], cancer, coronary heart disease and all-cause mortality [2]. Obesity has been associated with reduced physical HRQoL in numerous studies [3, 4] with a stronger association in women compared to men [5–8]. Several recent studies have demonstrated that even a “metabolically healthy obese” phenotype, i. e. persons with obesity in the absence of metabolic risk factors or comorbidities, show reduced scores of physical HRQoL compared to the group of metabolically healthy non-obese (MHNO) [9–11]. However, these previous studies applied different definitions of metabolic health and HRQoL and considered various subsets of potential confounders. Potential confounders of the association between obesity and lower HRQoL include higher age, low educational status, health-related behaviours [12], and chronic health conditions [13]. In general, metabolic health is associated with higher HRQoL, however, sex-specific associations are still unclear [14]. Previous studies described similar effects among men and women [15] as well as different associations among both sexes, with a negative effect of an impaired health status observed only among women [14]. To our knowledge only one previous study on obesity among metabolic health men and women conducted sex-specific analyses and found that metabolic health had a greater impact on HRQoL than obesity in men while similar effects for metabolic health and obesity were seen in women [9].

Against this background the aim of this study was (1) to determine whether obesity is related to lower HRQoL independent of metabolic health status also considering age, low educational status, health-related behaviours, and comorbidities as potential confounders, and (2) whether associations are similar among men and women.

## Methods

### Study design and sample

The German Health Interview and Examination Survey for Adults 2008–11 (DEGS1) is part of the German health monitoring system. Participants were selected from local population registries in a two-stage sampling procedure. A sample of 7115 participants had participated in the examination part and within this sample representative cross-sectional analyses for the age range of 18–79 years are possible. Exclusion of participants with missing data on body mass index (BMI) and metabolic health status resulted in a study sample of 6860 participants (3298 men, 3562 women). The design of DEGS1 has been previously described in detail [16].

### Data collection and study variables

#### Physical health-related quality of life

A validated self-administered German language version [17] of the Medical Outcome Short Form-36 version 2 (SF-36 version 2) [18, 19] was used to measure physical HRQoL. The questionnaire comprises 36 questions covering eight domains: physical functioning, role-physical, bodily pain, general health, vitality, social functioning, role-emotional and mental health [20]. To improve international comparability “norm-based scoring” was conducted; therefore, the scales were z-transformed using the average values and standard deviations of the 1998 American normative random sample [21]. Two summary measures, the physical component summary (PCS) and the mental component summary combine the information of the eight scales. The present analysis focussed on the PCS score, which correlates mostly with the domains physical functioning, role-physical and bodily pain [20].

#### Metabolic health and obesity categories

Body weight, body height and waist circumference (WC) were measured by trained staff [22]. Body weight was measured using a calibrated electronic scale (SECA, column scale 930, Hamburg, Germany) with a precision of 0.1 kg and body height with a portable stadiometer (Holtain Ltd., UK) with a precision of 0.1 cm. BMI was calculated as body weight divided by height squared and categorized as non-obese (BMI < 30 kg/m^2^) and obese (BMI ≥ 30 kg/m^2^) [23]. WC was measured at the minimal waist using a flexible, non-stretchable tape. Among participants with no visible waist, WC was measured at the midpoint between the lowest rib and the ileac crest. Blood pressure was measured on the right arm following a standardized protocol and using an automated oscillometric device (Datascope Accutorr Plus) [24]. Self-reported physician-diagnosed diseases were obtained in computer-assisted interviews administered by specifically trained physicians [16]. All medications taken in the 7 days prior to the interview were documented by trained medical staff and coded according to the Anatomical Therapeutic Chemical classification system [25]. Blood samples were taken and time of blood draw and number of hours since last meal were recorded [16]. Fasting time was calculated and considered for serum triglyceride cut-off definitions as applied in previous studies [26, 27]. Laboratory analyses were conducted to determine glycated hemoglobin (HbA1c; immunoturbidimetric method, Architect ci8200), triglycerides (enzymatic procedure, Architect ci8200) and high density lipoprotein cholesterol (HDL-C; enzymatic procedure, Architect ci8200).

Metabolic health was defined based on the ATPIII definition [28] as described before for this study population [27] and as fulfilling three or more of the following criteria:

- WC < 88 cm in women and WC < 102 cm in men
- HbA1c < 5.7% and had no drug treatment and no physician-diagnosed diabetes
- Blood pressure < 130/85 mmHg and had no drug treatment and no physician-diagnosed hypertension
- Triglycerides < 1.7 mmol/l (or < 2.1 mmol/l among participants, who fasted less than 8 h) and had no drug treatment and no physician-diagnosed dyslipidemia
- HDL-C ≥ 1.0 mmol/l in men and ≥ 1.3 mmol/l in women

Categories of metabolic health status and obesity were defined as metabolically healthy non-obese (MHNO), metabolically unhealthy non-obese (MUNO), metabolically healthy obese (MHO), and metabolically unhealthy obese (MUO).

### Covariables

Covariables were selected to adjust for potential confounders as described in previous studies [9–11]. Educational status (low, middle, high), smoking (no, occasionally, daily), physical activity ≥ 2.5 h per week (no, yes) and alcohol consumption (never: 0 g, moderate: 0- < 10 g in women and 0- < 20 g in men, high: ≥ 10 g in women and ≥ 20 g in men) was assessed by self-administered questionnaires. Comorbidity was defined as reporting at least one of the following physician-diagnosed conditions in personal computer-assisted interviews: cancer, myocardial infarction, stroke asthma, coronary heart disease, chronic heart failure, asthma, musculoskeletal conditions. Based on a question asking for additional diseases requiring current treatment, self-reported gallbladder disease was considered as an obesity-related health condition. Detailed information regarding the definition of covariables is shown in Additional file 1.

### Analysis

In descriptive analyses means, percentages and 95% confidence intervals were calculated in strata of metabolic health status and obesity categories. All analyses were stratified for sex. Linear regression analyses were performed with the PCS as dependent variable and categories of metabolic health and obesity as independent variable (Model 1). The group of MHNO was used as the reference category. Model 2 was additionally adjusted for age (squared). Model 3 additionally included alcohol consumption, educational status, physical activity, smoking, and comorbidity. In a sensitivity analysis linear regression analyses were performed with the physical functioning score as dependent variable because the association between BMI and HRQoL seems to be most pronounced in this domain [29]. In linear regression analyses gender differences in PCS were identified in categories of metabolic health and obesity. The level of statistical significance was set at *p* = 0.05 based on two-sided tests. SAS 9.4 survey procedures (SAS Institute Inc., Cary, NC) were used for all statistical analyses. To account for the clustered survey design specific survey procedures were used. Analyses were weighted using a weighting factor to correct for deviations of the net sample from the population structure in Germany and compensate for stratification.

## Results

Table 1 (men) and Table 2 (women) present the study characteristics by strata of metabolic health status and obesity. In men the distribution of participants was MHNO (*n* = 1900), MUNO (*n* = 608), MHO (*n* = 196), MUO (*n* = 594) and in women it was MHNO (*n* = 2193), MUNO (*n* = 514), MHO (*n* = 277), MUO (*n* = 578). MHNO (mean age men: 42.1 years, women: 42.4 years) were younger than MHO and MUO; MUNO (men: 55.6 years, 59.3 years) was the oldest group. Percentage of low educational status was lowest in MHNO (men: 30.7%, women: 27.8%) and highest in MUO (men: 51.3%, women: 59.5%). Percentages of daily smoking and physical activity ≥ 2.5 h were lower among metabolically unhealthy men and women. Percentages of high alcohol consumption were highest among MUNO for men (22.7%) and highest among MHNO for women (14.9%). The prevalence of comorbidities was higher among metabolically unhealthy men and women. The PCS was highest among MHNO (men: 54.0, women: 53.1) and lowest among MUO (men: 47.0, women: 44.1).

**Table 1:** Study characteristics among men (mean/% and 95% confidence interval)

	N missing	MHNO *N* = 1900	MUNO *N* = 608	MHO *N* = 196	MUO *N* = 594
Age (mean, years)	0	42.1 (41.3–42.9)	56.8 (55.2–58.4)	44.4 (42.3–46.6)	55.6 (54.2–57.1)
Educational status (%)	21				
Low		30.7 (27.5–34.0)	41.3 (35.8–47.0)	41.2 (32.3–50.7)	51.3 (45.5–57.2)
Middle		51.3 (48.4–54.2)	38.8 (33.9–43.8)	48.3 (39.3–57.4)	38.3 (32.7–44.1)
High		18.0 (15.9–20.4)	20.0 (16.0–24.6)	10.5 (6.7–16.0)	10.4 (8.0–13.5)
Smoking (%)	17				
No		64.1 (60.9–67.2)	74.0 (68.1–79.2)	66.8 (57.0–75.4)	72.1 (66.7–76.9)
Occasionally		7.5 (6.2–9.1)	4.3 (2.5–7.3)	5.9 (3.4–10.3)	5.6 (3.6–8.7)
Daily		28.4 (25.6–31.4)	21.7 (17.0–27.2)	27.3 (18.9–37.6)	22.3 (17.9–27.6)
Physical activity ≥ 2.5 h (%)	116	28.2 (25.5–31.0)	18.4 (14.6–22.9)	30.0 (22.5–38.6)	18.9 (15.0–23.4)
Alcohol consumption (%)	55				
Never		10.4 (8.4–12.8)	8.4 (5.8–11.9)	11.3 (6.7–18.3)	9.2 (6.2–13.3)
Moderate		72.0 (69.1–74.8)	68.9 (64.9–72.7)	72.6 (63.7–80.0)	71.5 (65.9–76.4)
High		17.6 (15.6–19.8)	22.7 (19.3–26.5)	16.1 (10.7–23.4)	19.4 (15.4–24.1)
One or more comorbidities (%)	29	25.7 (23.2–28.3)	51.9 (46.8–57.0)	25.7 (23.2–28.3)	49.5 (44.1–54.9)
Physical component summary (mean)^a^	158	54.0 (53.6–54.5)	49.8 (48.8–50.9)	51.8 (50.4–53.3)	47.0 (45.9–48.2)

*MHNO* metabolically healthy non-obese, *MUNO* metabolically unhealthy non-obese, *MHO* metabolically healthy obese, *MUO* metabolically unhealthy obese

^a^Physical Health Related Quality of Life according to SF36v2

**Table 2:** Study characteristics among women (mean/% and 95% confidence interval)

	N missing	MHNO *N* = 2193	MUNO *N* = 514	MHO *N* = 277	MUO *N* = 578
Age (mean, years)	0	42.4 (41.7–43.1)	61.9 (60.6–63.3)	47.0 (45.0–48.9)	59.3 (57.8–60.8)
Educational status (%)	17				
Low		27.8 (25.3–30.6)	55.9 (49.9–61.8)	45.1 (37.0–53.4)	59.5 (53.8–65.0)
Middle		56.2 (53.3–59.1)	36.0 (30.3–42.1)	49.9 (42.0–57.9)	36.5 (31.4–41.9)
High		16.0 (13.6–18.6)	8.1 (6.1–10.7)	5.0 (3.1–8.0)	4.0 (2.5–6.2)
Smoking (%)	17				
No		69.9 (67.0–72.7)	78.9 (73.4–83.5)	74.5 (67.8–80.3)	79.1 (74.3–83.2)
Occasionally		7.7 (6.4–9.2)	2.1 (1.1–4.1)	4.1 (2.1–7.7)	1.5 (0.7–3.3)
Daily		22.4 (20.0–25.0)	19.0 (14.8–24.1)	21.4 (16.1–27.9)	19.4 (15.5–24.1)
Physical activity ≥ 2.5 h (%)	110	15.9 (14.1–18.0)	13.7 (10.3–18.1)	16.9 (12.0–23.2)	14.0 (10.5–18.4)
Alcohol consumption (%)	44				
Never		16.3 (14.5–18.3)	17.0 (13.0–21.8)	21.0 (15.6–27.8)	29.7 (25.1–34.7)
Moderate		68.8 (66.2–71.3)	70.8 (65.3–75.8)	68.9 (60.7–76.1)	61.1 (55.8–66.1)
High		14.9 (13.1–16.9)	12.2 (8.8–16.8)	10.0 (5.5–17.4)	9.2 (6.2–13.6)
One or more comorbidities (%)	22	26.0 (23.7–28.6)	54.6 (49.4–59.7)	40.2 (34.0–46.7)	59.9 (54.7–64.9)
Physical component summary (mean)^a^	150	53.1 (52.7–53.6)	47.6 (46.4–48.7)	49.2 (47.8–50.6)	44.1 (43.1–45.1)

*MHNO* metabolically healthy non-obese, *MUNO* metabolically unhealthy non-obese, *MHO* metabolically healthy obese, *MUO* metabolically unhealthy obese

^a^Physical Health Related Quality of Life according to SF36v2

Table 3 shows the PCS in relation to categories of metabolic health and obesity for men and women. Additionally, Table S1 (Additional file 2) presents the estimations for physical functioning. Compared with MHNO, all other categories of metabolic health and obesity were consistently associated with lower PCS values among men and among women. The inverse association with PCS was strongest for MUO (men: −7.0, women: −9.0), intermediate for MUNO (men: −4.2, women: −5.6) and least pronounced for MHO (men: −2.2, women −3.9. Adjusting for age (Model 2) and further adjustment for sociodemographic characteristics, lifestyle variables and comorbidities (Model 3) decreased the association among all strata of metabolic health and obesity. After adjusting for confounding variables, the effect was strongest for MUO (men: −3.9, women: −4.9), intermediate for MHO (men: −1.8, women: −2.1) and least pronounced for MUNO (men: −1.3, women: −1.5). The results for the PCS were similar to the results found in sensitivity analyses using the physical functioning score as dependent variable (Additional file 2: Table S1).

**Table 3:** Linear regression models for physical component summary (SF-36 v2) according to metabolic health and obesity status

	N	MHNO	MUNO				MHO				MUO			
			Beta	Lower CL	Upper CL	*p*	Beta	Lower CL	Upper CL	*p*	Beta	Lower CL	Upper CL	*p*
Men														
Model 1	3140	Reference	−4.2	−5.3	−3.1	<.001	−2.2	−3.6	−0.8	.003	−7.0	−8.2	−5.8	<.001
Model 2	3140	Reference	−1.7	−2.7	−0.6	.002	−1.9	−3.3	−0.5	.006	−4.7	−5.9	−3.5	<.001
Model 3	2988	Reference	−1.3	−2.3	−0.3	.014	−1.8	−3.1	−0.5	.009	−3.9	−5.1	−2.7	<.001
Women														
Model 1	3412	Reference	−5.6	−6.8	−4.4	<.001	−3.9	−5.4	−2.5	<.001	−9.0	−10.2	−7.9	<.001
Model 2	3412	Reference	−2.1	−3.3	−0.9	<.001	−3.3	−4.7	−1.9	<.001	−6.2	−7.4	−5.0	<.001
Model 3	3270	Reference	−1.5	−2.7	−0.3	.018	−2.1	−3.2	−1.0	<.001	−4.9	−6.1	−3.6	<.001

Model 1: unadjusted

Model 2: adjusted for age (squared)

Model 3: additionally adjusted for educational status, smoking, physical activity, alcohol consumption, comorbidities

*CL* confidence limit, *MHNO* metabolically healthy non-obese, *MUNO* metabolically unhealthy non-obese, *MHO* metabolically healthy obese, *MUO* metabolically unhealthy obese

Table 4 presents gender differences in PCS for categories of metabolic health and obesity status. PCS scores were consistently lower among women compared to men in all strata. Gender differences were lowest among MHNO (−0.9) and highest among MUO (−2.9). After adjusting for confounding variables differences remained statistically significant only among MHNO.

**Table 4:** Gender differences (reference = men) in physical component summary (SF-36 v2) in categories of metabolic health and obesity status

	MHNO *N* = 3945				MUNO *N* = 1064				MHO *N* = 457				MUO *N* = 1084			
	Beta	Lower CL	Upper CL	*p*	Beta	Lower CL	Upper CL	*p*	Beta	Lower CL	Upper CL	*P*	Beta	Lower CL	Upper CL	*p*
Model 1	−0.9	−1.5	−0.2	.007	−2.3	−3.7	−0.8	.003	−2.7	−4.8	−0.5	.014	−2.9	−4.5	−1.4	<.001
Model 2	−0.8	−1.5	−0.2	.008	−1.1	−2.4	0.2	.084	−2.1	−4.2	−0.1	.043	−2.3	−3.7	−0.8	.003
Model 3	−0.8	−1.4	−0.1	.019	−1.0	−2.4	0.3	.130	−1.2	−3.1	0.6	.200	−1.1	−2.6	0.5	.180

Model 1: unadjusted

Model 2: adjusted for age (squared)

Model 3: additionally adjusted for educational status, smoking, physical activity, alcohol consumption, comorbidities

## Discussion

Obesity and metabolic aberration alone and in combination were associated with impaired physical HRQoL in both sexes. The effect of both factors in combination corresponds approximately to the effects for obesity and metabolic aberration alone. These results underline previous findings suggesting that MHO individuals are not always completely healthy with regard to HRQoL [10, 11] as well as morbidity of cardiovascular diseases [30, 31] and all-cause mortality [32, 33]. A recent clinical study found similar levels of HRQoL among MHO and MUO individuals [34]. This finding seems to contrast with our results with larger effects of MUO vs. MHNO compared to MHO vs. MHNO. The discrepancies may be caused by the clinical setting as patients who are willing to be treated probably are likely to suffer from obesity. However, in conclusion both studies support the finding that MHO is not a healthy state.

The association between physical HRQoL, metabolic health and obesity categories is stronger among women, as lower PCS values were found in all strata of metabolic health and obesity among women compared to men. Furthermore, differences between strata were greater among women than among men. Lower values of physical HRQoL among women compared to men were also reported in previous studies analysing the association with obesity [7, 8] and obesity in combination with metabolic health [9, 10] opposed to one meta-analysis where no gender differences were found [3]. In the present study, adjustment for potential confounders including education, health-related behaviours and comorbidities gender differences were attenuated. Thus, lower HRQoL among women than men might be partially explained by sex-differences in the prevalence of obesity-related comorbidities [35] and health-related behaviours e.g. physical activity [36]. Among both, men and women physical HRQoL was highest for the MHNO group followed by MHO and MUNO and lowest for the group of MUO. This is in contrast to findings of a Korean cross-sectional study [9], where HRQoL were measured using the euroqol-5 dimensions questionnaire. Among Korean men physical HRQoL was lowest among the group of MUNO, suggesting that an unhealthy metabolic status have a greater impact than obesity. In this previous study, no differences in physical HRQoL were observed between groups of metabolic status and obesity among men after adjusting for age, sociodemographic variables, lifestyle and comorbidity. Among Korean women, the results were similar to the findings of our study.

Persons in the different categories of obesity and metabolic health status substantially differed according to mean age, educational status and prevalence of one or more comorbidities. Previous studies reported lower values of HRQoL at higher age, among persons with chronic diseases, and in low educational status groups [13, 21, 37, 38]. Considering these potential confounders in multivariable regression analyses in the present study reduced the effect of lower physical HRQoL among all strata of metabolic health and obesity status compared to MHNO, but remained statistically significant among both sexes. A difference of two to three points in the PCS is considered clinically relevant [20]. Thus, after adjusting for confounding variables lower values of physical HRQoL among MHO compared to MHNO remained clinically relevant only among women. Adjustment for confounding factors had the largest impact among MUNO as differences in comparison to MHNO do not remain clinically significant.

The strength of this study is its large sample of the general population which allows stratification for gender and adjustment for potential confounding variables. Furthermore, anthropometric parameters were measured by standard protocols and trained staff and valid data collection tools were used to assess physical HRQoL, diseases, and various health determinants, and biological parameters. A few limitations have to be acknowledged. Since this is a cross-sectional study the direction of the causal pathway between physical HRQoL and metabolic health according to obesity category cannot be identified. Poorer physical HRQoL, characterized by sedentary behaviour, might increase weight gain and vice versa. Due to the study design we used different cut-offs among fasting and non-fasting participants for serum glucose and triglycerides. Finally, bias due to misclassification cannot be ruled out completely.

## Conclusion

Obesity was significantly related to lower physical HRQoL, independent of metabolic health status, which underlines previous findings that MHO status is not necessarily a healthy state. This inverse relationship can be partly explained by age, educational status, health-related behaviors, and comorbidities. The associations were found in both sexes and were consistently more pronounced among women compared to men.

## Additional files

Additional file 1: Covariables (PDF 160 kb).

Additional file 2: Sensitivity analyses with physical functioning as dependent variable (PDF 21 kb).

## Abbreviations

BMI: body mass index
HRQoL: health-related quality of life
MHNO: Metabolically healthy non-obese
MHO: Metabolically healthy obese
MUNO: Metabolically unhealthy non-obese
MUO: Metabolically unhealthy obese
PCS: physical component score
WC: waist circumference
