# Supplementary material for: Physical health-related quality of life in relation to metabolic health and obesity among men and women in Germany
Source: Health Qual Life Outcomes. 2017 Jun 10;15:122. doi: 10.1186/s12955-017-0688-7 (PMC5466792; doi:10.1186/s12955-017-0688-7)
Supplement: Supplementary file 1 — Covariables (PDF 160 kb). [file 12955_2017_688_MOESM1_ESM.pdf]

Additional File 1: Covariables

Information on academic and professional qualification was used to determine the **educational status** [1]. The Comparative Analysis of Social Mobility in Industrial Nations (CASMIN) classification system [2] was used for classification into three groups: “low”, “medium”, and “high”.

Smoking was assed using four categories (“yes, daily”, “yes, occasionally”, “ex-smoker”, “never”) [3]. For the present analyses, **current smoking** was categorized as “no”, “occasionally”, and “daily”.

The questions regarding **physical activity** referred to the last three months [4]. The participants were asked how many days per week and how long they are physically active on average. The information obtained from both questions was combined to estimate the proportion of those who fulfil the WHO recommendation of at least 2.5 hours per week [5].

A semi-quantitative self-administered food frequency questionnaire was used to assess frequency and portion size of non-alcoholic-beer, beer, wine, spirits, cocktails (during the last 4 weeks). Daily alcohol consumption was calculated by summing the alcohol content of these beverages. **Alcohol consumption** was defined as: never (0g), moderate: (0-≤ 10g in women and 0-≤ 20g in men), and high (> 10g in women and > 20g in men) [6].

Previous physician-diagnosis of cancer, myocardial infarction, stroke asthma, coronary heart disease, chronic heart failure, asthma and musculoskeletal conditions were assessed using a standardized computer-assisted personal interview [7]. Trained study physicians asked the participants whether a physician had ever told them that they had the respective disease or health problem. Furthermore, participants were asked for additional diseases requiring current treatment. **Comorbidity** was defined as reporting at least one of the physician-diagnosed conditions or self-reported gallbladder disease.

1. Lampert T, Kroll LE, Müters S, Stolzenberg H: **Measurement of socioeconomic status in the German Health Interview and Examination Survey for Adults (DEGS1).** Epidemiologie und Gesundheitsberichterstattung; 2013.
2. Brauns H, Scherer S, Steinmann S: **The CASMIN educational classification in international comparative research.** In *Advances in cross-national comparison.* Edited by Hoffmeyer-Zlotnik J, Wolf C. New York: Kluwer; 2003: 221-244
3. Lampert T, Lippe Evd, Müters S: **Prevalence of smoking in the adult population of Germany - Results of the German Health Interview and Examination Survey for Adults (DEGS1).** Epidemiologie und Gesundheitsberichterstattung; 2013.
4. Krug S, Jordan S, Mensink G, Müters S, Finger J, Lampert T: **Physical activity - Results of the German Health Interview and Examination Survey for Adults (DEGS1).** Epidemiologie und Gesundheitsberichterstattung; 2013.
5. World Health Organization: **Global recommendations on physical activity for Health.** (WHO ed. Geneva; 2010.
6. Truthmann J, Busch MA, Scheidt-Nave C, Mensink GB, Gosswald A, Endres M, Neuhauser H: **Modifiable cardiovascular risk factors in adults aged 40-79 years in Germany with and without prior coronary heart disease or stroke.** *BMC Public Health* 2015, **15:**701.
7. Scheidt-Nave C, Kamtsiuris P, Gößwald A, Hölling H, Lange M, Busch MA, Dahm S, Dölle R, Ellert U, Fuchs J, et al: **German health interview and examination survey for adults (DEGS) - design, objectives and implementation of the first data collection wave.** *BMC Public Health* 2012, **12:**1-16.
