# Supplementary material for: Physical health-related quality of life in relation to metabolic health and obesity among men and women in Germany
Source: Health Qual Life Outcomes. 2017 Jun 10;15:122. doi: 10.1186/s12955-017-0688-7 (PMC5466792; doi:10.1186/s12955-017-0688-7)
Supplement: Supplementary file 2 — Sensitivity analyses with physical functioning as dependent variable (PDF 21 kb). [file 12955_2017_688_MOESM2_ESM.pdf]

Additional File 2: Sensitivity analyses with physical functioning as dependent variable

TabS1: Linear regression models for physical functioning (SF-36 v2) according to metabolic health and obesity status

	N	MHNO	MUNO				MHO				MUO			
			Beta	Lower CL	Upper CL	p	Beta	Lower CL	Upper CL	p	Beta	Lower CL	Upper CL	p
**Men**														
Model1	3189	Reference	-3.3	-4.3	-2.3	**<.001**	-2.1	-3.3	-0.9	**.001**	-6.0	-7.1	-5.0	**<.001**
Model2	3189	Reference	-1.2	-3.1	-0.7	**.016**	-1.9	3.1	-0.7	**.002**	-4.1	-5.1	-3.1	**<.001**
Model3	3030	Reference	-0.9	-1.8	0.5	.064	-1.6	-2.8	-0.5	**.007**	-3.2	-4.2	-2.1	**<.001**
**Women**														
Model1	3476	Reference	-5.7	-6.9	-4.5	**<.001**	-3.9	-5.3	-2.6	**<.001**	-9.0	-10.1	-7.9	**<.001**
Model2	3476	Reference	-2.4	-3.6	-1.2	**<.001**	-3.4	-4.7	-2.1	**<.001**	-6.3	-7.5	-5.1	**<.001**
Model3	3326	Reference	-1.7	-2.9	-0.5	**.005**	-2.1	-3.2	-1.1	**<.001**	-5.1	-6.3	-3.9	**<.001**

CL: confidence limit, MHNO: metabolically healthy non-obese, MUNO: metabolically unhealthy non-obese, MHO: metabolically healthy obese, MUO: metabolically unhealthy obese

Model1: unadjusted

Model2: adjusted for age (squared)

Model3: additionally adjusted for educational status, smoking, physical activity, alcohol consumption, comorbidities
